# Association of childhood health with adulthood chronic kidney disease: results from the China Health and Retirement Longitudinal Study

**DOI:** 10.3389/fpubh.2025.1538744

**Published:** 2025-04-22

**Authors:** Rong Lian, Zheng-he Wang

**Affiliations:** ^1^Department of Nephrology, Guangzhou First People’s Hospital, The Second Affiliated Hospital, School of Medicine, South China University of Technology, Guangzhou, China; ^2^Department of Epidemiology, School of Public Health, Southern Medical University, Guangzhou, China

**Keywords:** childhood health, health status, Glomerular Filtration Rate, chronic kidney disease, community child health, nephrology, public health, kidney and urinary tract disorders

## Abstract

**Objective:**

Adverse Childhood Experiences have been well-documented as a risk factor for chronic kidney disease (CKD) in adulthood. However, the link between childhood health and adulthood CKD risk is still unclear. This study aimed to explore the connection between childhood health and the likelihood of developing CKD in adulthood.

**Methods:**

Participants were drawn from the third wave of the China Health and Retirement Longitudinal Study (CHARLS). The CKD was identified based on the estimated Glomerular Filtration Rate (eGFR) and self-reported doctor-diagnosed kidney disease. Childhood health status was assessed through a standard questionnaire and categorized into excellent, fair, and poor groups.

**Results:**

The prevalence of CKD was 11.7% (1,480 out of 12,609). The eGFR levels in the self-reported Fair and Poor groups were significantly lower than those in the Excellent group (*p* < 0.05). Compared to the Excellent group, individuals in the Poor group reported a higher risk of CKD (*OR* = 1.38; 95% *CI*: 1.12–1.70; *p* = 0.002), even after adjusting for factors such as age, sex, smoking, alcohol consumption, physical activity, highest education level, use of Chinese traditional medicine, diabetes, hypertension, BMI, marital status, and annual household income (*OR* = 1.24; 95% *CI*: 1.01–1.54; *p* = 0.047).

**Conclusion:**

The CKD prevalence is notably high in the Chinese adults aged more than 45 years, and a history of poor health in childhood may significantly contribute to the risk of CKD in later life.

## Introduction

Chronic kidney disease (CKD) involves long-term structural and functional changes in the kidneys resulting from a variety of underlying causes (1). It has become a significant public health issue. The prevalence of CKD has been steadily increasing, which has placed a considerable burden on healthcare systems. The disease’s impact is not only limited to individuals but also extends to society due to the associated healthcare costs and loss of productivity. Various factors contribute to the rise in CKD cases, including the growing prevalence of diabetes and hypertension, which are key risk factors for kidney damage. The global focus on CKD underscores the need for effective preventive measures, early diagnosis, and management strategies to mitigate its impact (2, 3). In China, CKD has become notably prevalent among adults, reflecting a concerning trend in the country’s public health landscape. Data from 2019 reveals that there were approximately 150.5 million cases of CKD, representing 10.6% of the adult population. This figure highlights the widespread nature of the disease and its significant impact on the Chinese population. Additionally, there were 196,726 deaths attributed to CKD in the same year. Looking ahead, projections suggest that the prevalence of CKD in China will increase to 11.7% by 2029, with mortality rates rising to 17.1 per 100,000 individuals (4). These trends emphasize the urgent need for comprehensive strategies to address and manage CKD effectively in the coming years.

Factors of CKD are extremely complex. Well-documented major factors contributing to various health conditions encompass a range of genetic, physiological, and lifestyle elements. Except for these factors mentioned above, recent life-course epidemiological studies suggest 15–20% of adult CKD risk may originate in early developmental periods (5). The Developmental Origins of Health and Disease (DOHaD) framework, pioneered by Barker and colleagues (6), posits that developmental plasticity during critical early-life periods allows organisms to adapt to suboptimal environments (e.g., malnutrition, psychosocial stress) through persistent metabolic and epigenetic reprogramming - a survival advantage evolutionarily that paradoxically elevates susceptibility to cardiorenal diseases in later life (7). Substantial evidence links specific developmental insults (e.g., intrauterine growth restriction, childhood malnutrition) with reduced nephron endowment and accelerated renal function decline (8, 9). Nevertheless, current DOHaD-CKD epidemiology predominantly relies on extreme-exposure cohorts, particularly famine studies (Dutch Hunger Winter; Chinese Great Famine) that quantify severe but transient malnutrition (10, 11). While these provide mechanistic insights, they represent “natural experiments” with limited generalizability to contemporary populations experiencing moderate yet chronic developmental adversities - a critical gap given rising childhood obesity/micronutrient deficiencies in transitional economies. This study innovatively employs self-reported childhood health as a composite exposure metric capturing nutritional, infectious, and psychosocial stressors across development. Although retrospective recall may introduce non-differential misclassification, this approach enables cost-effective assessment of developmental origins in settings lacking longitudinal biomedical data - a methodological advance for DOHaD research in resource-constrained regions.

Self-reported childhood health is a key indicator of early-life health and a crucial element of adverse childhood experiences (ACEs). Studies have found that poor self-reported childhood health correlates with the number of adverse childhood experiences reported (12). Growing evidence has linked ACEs to conditions such as hypertension (13), diabetes (14), cardiovascular diseases (15, 16). However, whether self-reported childhood health predicts adult CKD incidence independent of traditional risk factor remains unclear, which was important for the DOHaD framework, highlighting how subjective childhood health perceptions may capture synergistic biological and socioeconomic pathways to CKD risk.

This study uses data from wave 3 of the China Health and Retirement Longitudinal Study (CHARLS) to examine whether self-reported childhood health predicts adult CKD incidence independent of traditional risk factors, while also assessing potential gender differences in this association, which could inform life-course prevention strategies by identifying at-risk populations through simple retrospective screening.

## Materials and methods

### Study design and participants

The participants for this cross-sectional study were drawn from the third wave of the China Health and Retirement Longitudinal Study (CHARLS). The design and sampling methodology of CHARLS have been detailed in prior publications (17). The follow-up surveys were conducted in 2013, 2015, and 2018. This study utilized data from the third wave follow-up survey, carried out from July 1 to September 30, 2015, when the most recent blood indicator measurements were available. CHARLS was approved by the institutional review board of Peking University (IRB00001052-11015), and written informed consent was obtained from all participants.

**Figure 1:**
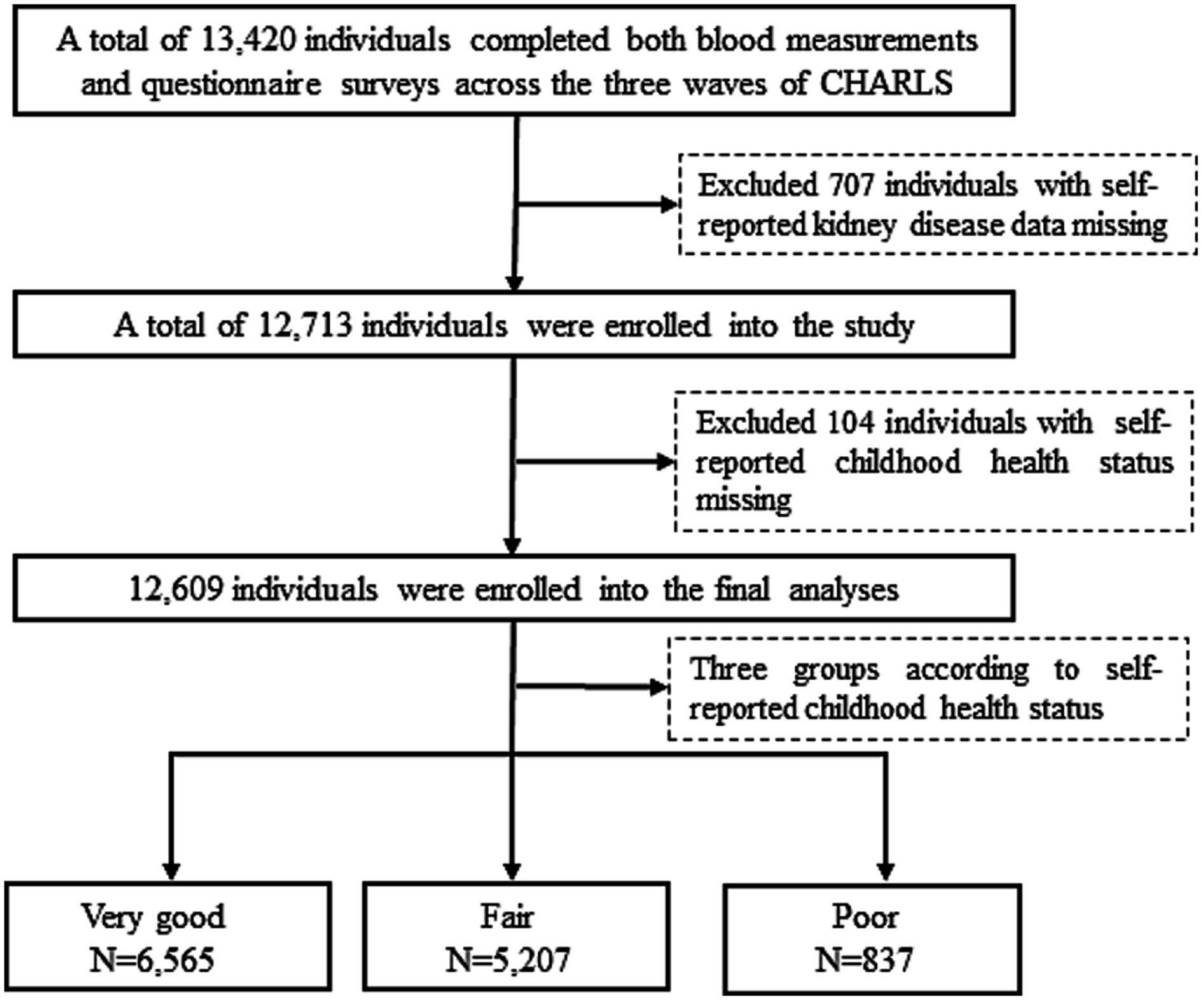
Flowchart of the study population.

Out of 13,420 participants who completed both blood measurements and questionnaire surveys across the three waves of CHARLS, 707 participants were excluded due to missing self-reported data on kidney disease (excluding tumors or cancers), and 104 were excluded for missing self-reported childhood health status. Thus, the final sample for this study comprised 12,609 participants (Figure 1).

### Definition of childhood health status

Childhood health status was assessed using self-reported data collected through face-to-face interviews. Participants’ health status during childhood, up to and including age 15, was categorized into three distinct groups: Excellent, Fair, and Poor. This categorization was based on responses to the question, “How would you evaluate your health during childhood, up to and including age 15? Excellent, very good, good, fair, poor?” Participants who reported “Excellent” or “Very good” were classified as having Excellent health. Those who reported “Good” or “Fair” were categorized as having Fair health, while those who reported “Poor” were classified as having Poor health.

### Definition of CKD

Chronic Kidney Disease (CKD) is characterized by elevated blood creatinine levels and self-reported kidney disease diagnosed by a physician. The estimated Glomerular Filtration Rate (eGFR) was determined using the 2009 Chronic Kidney Disease Epidemiology Collaboration (CKD-EPI) equation (18). A reduced renal function was defined as an eGFR of less than 60 mL/min per 1.73 m^2^.

### Covariates

Age was determined by subtracting the birthdate from the survey date and was subsequently categorized into five distinct age groups: 45–54 years, 55–64 years, 65–74 years, and 75 years and older. Educational attainment was assessed via questionnaires and classified into the following categories: primary education or less, junior high school, senior and above. Information regarding the use of Traditional Chinese Medicine (TCM) was obtained through a questionnaire. Participants were classified as using TCM if they responded affirmatively to the question: “Are you currently using Traditional Chinese Medicine to treat hypertension, dyslipidemia, diabetes, cancer, chronic lung diseases, liver disease, or their complications?” Body Mass Index (BMI) was calculated by dividing weight (in kilograms) by the square of height (in meters) and was categorized into four groups: underweight (BMI < 18.5 kg/m^2^), normal weight (18.5 kg/m^2^ ≤ BMI ≤ 24.0 kg/m^2^), overweight (24.0 kg/m^2^ < BMI < 28.0 kg/m^2^), and obese (BMI ≥ 28.0 kg/m^2^). Smoking status was classified into three categories: never smoked (smoked ≤100 cigarettes in a lifetime), former smoker (smoked >100 cigarettes in a lifetime but quit more than 1 year ago), and current smoker (smoked at least one cigarette per day in the past year). Drinking status was similarly categorized into: never drank (never consumed alcohol in a lifetime), former drinker (consumed at least one unit per month but stopped more than 1 year ago), and current drinker (consumed at least one standard unit per month in the past year). Physical activity was assessed using the International Physical Activity Questionnaire Short Form (IPAQ-SF) and was categorized as light, moderate, or vigorous (19). Marital status was classified into: married with spouse present, and other statuses (including married but not living with spouse temporarily, separated, divorced, widowed, never married, and cohabiting). Participants’ annual household income was divided into quartiles, with groups designated as Q1 (<25th percentile), Q2 (25th-50th percentile), Q3 (50th-75th percentile), and Q4 (>75th percentile).

### Statistical analysis

Statistical analyses were conducted using Stata 15 and IBM SPSS 20.0 (IBM Corporation, Armonk, NY, United States). Continuous variables were summarized as mean ± standard deviation (SD), while categorical variables were expressed as percentages (%).

The relationship between self-reported health status during childhood and the risk of chronic kidney disease in adulthood was examined through binary univariate and multivariable logistic regression models, utilizing maximum likelihood estimation. Model 1 represented an unadjusted analysis, while Model 2 adjusted for age, sex, smoking, alcohol consumption, physical activity level, highest educational attainment, and use of Traditional Chinese Medicine (TCM). Model 3 further adjusted for diabetes, hypertension, body mass index (BMI) categories, marital status, and annual household income. Subsequent analyses were stratified by age group, sex, region, education level, TCM use, BMI category, diabetes, and hypertension. Additionally, interaction effects were investigated by incorporating multiplicative interaction terms into the logistic regression models.

## Results

**Figure 2:**
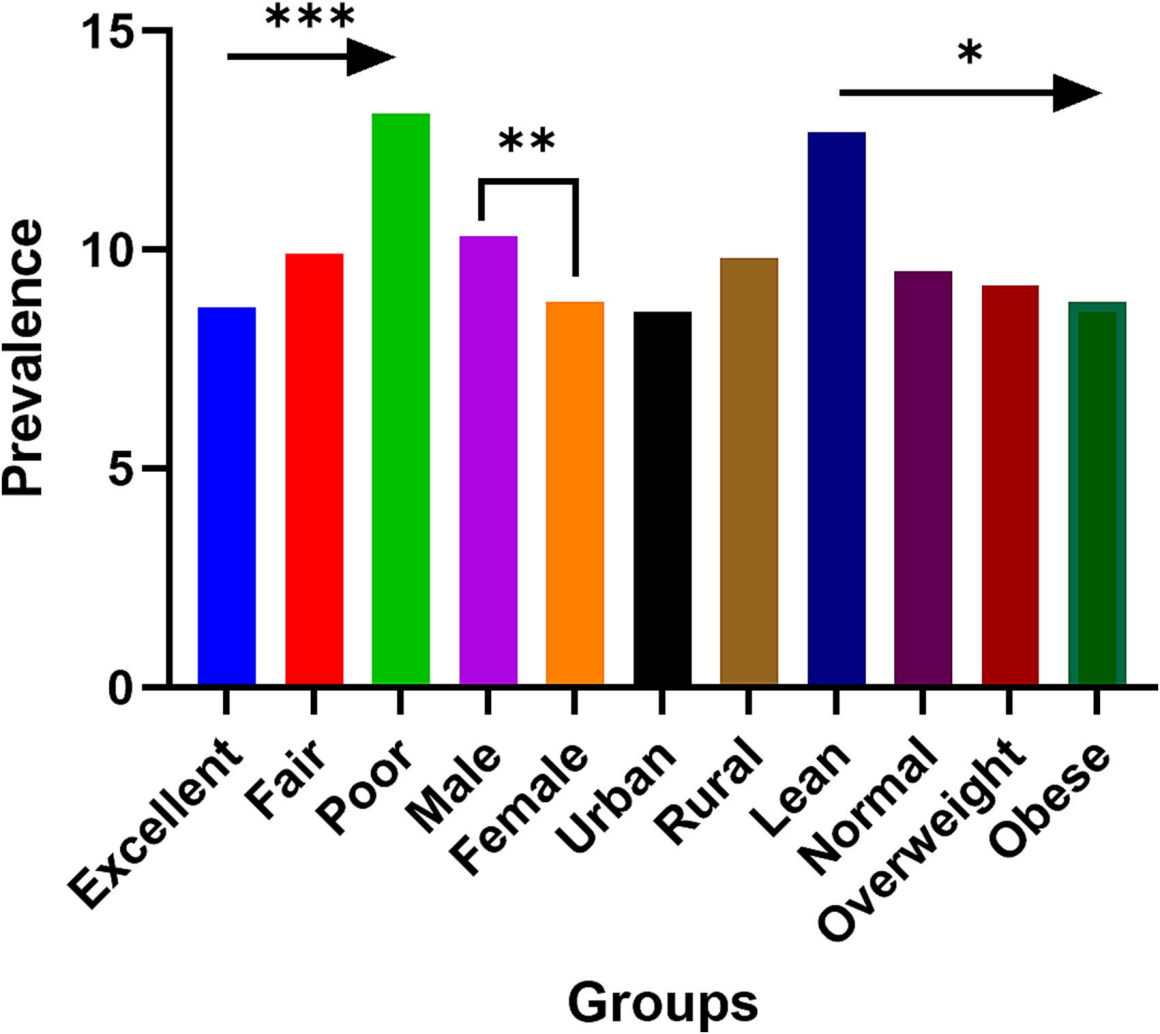
The prevalence of chronic kidney disease among participants with different self-reported childhood health status, sex, region, and nutrition condition. *p for trend<0.05; **p < 0.01; ***p for trend <0.001.

Table 1 provides a summary of the participants’ basic characteristics. The study included a total of 12,609 individuals, with a chronic kidney disease (CKD) prevalence of 11.7% (1,480/12,609) among Chinese adults. Analysis by self-reported childhood health status reveals a significant upward trend in CKD prevalence from those reporting excellent to poor childhood health (Figure 2). There were significant differences in the distribution of age groups, sex, region, traditional Chinese medicine (TCM) use, education level, smoking, drinking, BMI categories, hypertension, diabetes, marital status, and annual household income between participants with CKD and those without (*p* < 0.05). However, no significant difference in physical activity levels was observed between these groups (*p* = 0.352).

**Table 1:** The basic characteristics of participants.

Variables	Total (*n* = 12,609)	Normal (*n* = 11,129)	CKD (*n* = 1,480)	*p*-value
Age group				<0.001
45–49	3,849(30.5)	3,627(32.6)	222(15.0)	
50–59	4,095(32.5)	3,656(32.9)	439(29.7)	
60–69	3,332(26.4)	2,828(25.4)	504(34.1)	
70–79	1,333(10.6)	1,018(9.1)	315(21.3)	
Sex				0.001
Urban	5,872(46.6)	5,123(46.0)	749(50.6)	
Rural	6,737(53.4)	6,006(54.0)	731(49.4)	
Region				0.038
Urban	3,297(26.1)	2,943(26.4)	354(23.9)	
Rural	9,312(73.9)	8,186(73.6)	1,126(76.1)	
Education level				<0.001
Primary and below	9,016(71.5)	8,047(72.3)	969(65.5)	
Junior	2,371(18.8)	2,062(18.5)	309(20.9)	
Senior and above	1,222(9.7)	1,020(9.2)	202(13.6)	
Traditional medicine use				<0.001
No	10,380(82.3)	9,335(83.9)	1,045(70.6)	
Yes	2,229(17.7)	1,794(16.1)	435(29.4)	
Smoke				<0.001
Never smoke	7,453(59.1)	6,647(59.7)	806(54.5)	
Ever smoke	1,701(13.5)	1,438(12.9)	263(17.8)	
Current smoke	3,455(27.4)	3,044(27.4)	411(27.8)	
Drinking				0.003
Never drink	9,037(71.7)	7,963(71.6)	1,074(72.6)	
Ever drink	259(2.1)	213(1.9)	46(3.1)	
Current drink	3,313(26.3)	2,953(26.5)	360(24.3)	
PA level				0.352
Light PA	9,559(75.8)	8,419(75.6)	1,140(77.0)	
Middle PA	2,402(19.0)	2,128(19.1)	274(18.5)	
Vigorous PA	648(5.1)	582(5.2)	66(4.5)	
BMI category				<0.001
Underweight	669(5.3)	2,128(19.1)	274(18.5)	
Normal	6,059(48.1)	582(5.2)	66(4.5)	
Overweight	4,208(33.4)	556(5.0)	113(7.6)	
Obesity	1,673(13.3)	5,354(48.1)	705(47.6)	
Hypertension				<0.001
No	5,910(46.9)	5,298(47.6)	612(41.4)	
Yes	6,699(53.1)	5,831(52.4)	868(58.6)	
Diabetes				<0.001
No	10,047(80.7)	9,062(81.5)	985(74.3)	
Yes	2,403(19.3)	2,063(18.5)	340(25.7)	
Marital status				<0.001
Married with spouse present	10,538(83.6)	9,375(84.2)	1,163(78.6)	
Else	2,071(16.4)	1,754(15.8)	317(21.4)	
Annual household income				<0.001
Q1	3,152(25.0)	2,701(24.3)	451(30.5)	
Q2	3,152(25.0)	2,752(24.7)	400(27.0)	
Q3	3,152(25.0)	2,819(25.3)	333(22.5)	
Q4	3,153(25.0)	2,857(25.7)	296(20.0)	

CKD, chronic kidney disease; PA, physical activity; BMI, body mass index; TCM, traditional Chinese medicine. Q1, quartile 1; Q2, quartile 2; Q3, quartile 3; Q4, quartile 4.

Table 2 displays variations in the estimated Glomerular Filtration Rate (eGFR) across different childhood health statuses. The mean eGFR levels differed significantly among these statuses (*p* < 0.001). Both the Fair and Poor health groups exhibited lower eGFR levels compared to the excellent group. In gender-stratified analysis, females in the Fair group had significantly lower eGFR levels than those in the Excellent group (*p* = 0.007). Similar associations were observed when stratifying by region, smoking status, alcohol consumption, physical activity, diabetes, and hypertension.

**Table 2:** The level of estimated glomerular filtration rate (mL/min per 1·73 m^2^) among different childhood health status.

Variables	Total (*n* = 12,609)	Very good (*n* = 6,565)	Fair (*n* = 5,207)	Poor (*n* = 837)	*p*-value
Age group					
45–54	100.74 ± 16.15	101.62 ± 15.99	99.69 ± 16.36^***^	99.35 ± 15.81	<0.001
55–64	94.34 ± 15.80	94.27 ± 15.81	94.36 ± 16.03	94.67 ± 14.16	0.933
65–74	85.85 ± 17.34	86.06 ± 17.27	85.31 ± 17.56	87.37 ± 16.56	0.201
75+	75.24 ± 17.43	74.37 ± 17.82	76.10 ± 17.02	76.27 ± 16.99	0.243
Sex					
Male	95.23 ± 20.15	95.74 ± 20.40	94.68 ± 19.78	94.30 ± 20.21	0.106
Female	91.27 ± 16.27	91.88 ± 16.16	90.71 ± 16.67^*^	90.29 ± 14.29	0.007
Region					
Urban	91.76 ± 18.03	92.30 ± 18.25	91.12 ± 18.03	90.63 ± 15.38	0.145
Rural	93.59 ± 18.34	94.31 ± 18.46	92.92 ± 18.28^**^	92.40 ± 17.65^*^	0.001
Smoking status					
Never smoke	92.16 ± 17.05	93.04 ± 17.10	91.33 ± 17.27^***^	90.81 ± 14.94^*^	<0.001
Ever smoke	91.13 ± 20.13	91.04 ± 20.62	90.97 ± 19.78	93.00 ± 18.17	0.623
Current smoke	96.13 ± 19.50	96.39 ± 19.55	95.99 ± 19.15	94.63 ± 21.37	0.455
Drinking					
Never drink	91.91 ± 17.68	92.47 ± 17.76	91.29 ± 17.75^**^	91.32 ± 16.36	0.007
Ever drink	94.21 ± 19.84	92.23 ± 20.08	95.50 ± 20.18	101.43 ± 12.76	0.145
Current drink	96.30 ± 19.35	97.26 ± 19.56	95.59 ± 19.01	93.16 ± 19.28^**^	0.003
PA level					
Light PA	94.23 ± 18.31	95.16 ± 18.61	93.18 ± 18.08^*^	93.33 ± 16.86	0.011
Middle PA	93.00 ± 18.09	94.04 ± 18.08	92.06 ± 18.19^*^	90.28 ± 16.93	0.007
Vigorous PA	92.93 ± 16.99	93.66 ± 17.08	92.49 ± 17.29	90.22 ± 14.49	0.386
Diabetes					
No	93.39 ± 17.88	93.94 ± 17.90	92.84 ± 18.03^**^	92.48 ± 16.58	0.005
Yes	91.96 ± 19.83	92.93 ± 20.40	90.94 ± 19.05	90.17 ± 19.44	0.033
Hypertension					
No	95.24 ± 17.13	95.75 ± 17.19	94.63 ± 17.31	95.19 ± 15.32	0.059
Yes	91.24 ± 19.04	92.05 ± 19.23	90.53 ± 18.83^**^	88.86 ± 18.37^**^	<0.001
Total	93.11 ± 18.28	93.74 ± 18.42	92.49 ± 18.24^**^	92.02 ± 17.20^*^	<0.001

PA, physical activity. ^*^p < 0.05; ^**^p < 0.01; ^***^p < 0.001.

Table 3 details the relationship between self-reported childhood health status and CKD risk in adulthood. Relative to the Excellent group, the Fair (OR = 1.15; 95% CI: 1.02–1.28; *p* = 0.018) and Poor groups (OR = 1.38; 95% CI: 1.12–1.70; *p* = 0.002) exhibited higher CKD risk. Even after adjusting for age, sex, smoking, alcohol consumption, physical activity, highest education level, and TCM use (OR = 1.25; 95% CI: 1.01–1.54; *p* = 0.042), as well as additional adjustments for diabetes, hypertension, BMI category, marital status, and annual household income (OR = 1.24; 95% CI: 1.01–1.54; *p* = 0.047), the risk for the Poor group remained elevated by 25 and 24%, respectively.

**Table 3:** The association of childhood self-reported health status with adulthood CKD risk.

Health status	Model 1		Model 2		Model 3	
	OR(95%CI)	*p-*value	OR(95%CI)	*p-*value	OR(95%CI)	*p-*value
Good	Ref.		Ref.		Ref.	
Fair	1.15(1.02–1.28)	0.018	1.11(0.99–1.25)	0.080	1.10(0.98–1.24)	0.097
Poor	1.38(1.12–1.70)	0.002	1.25(1.01–1.54)	0.042	1.24(1.01–1.54)	0.047

OR, odds ratio; CI, confidence interval; Ref., reference. Model 1 was a crude model; Model 2 was adjusted for age, sex, region, smoking, alcohol consumption, physical activity, the highest education level, and TCM use; Model 3 was furthermore adjusted for diabetes and hypertension and BMI category, marital status, and annual household income.

Table 4 presents a stratified analysis of the association between childhood self-reported health status and adult CKD risk. In the age group of 55–64 years, the Poor group demonstrated a higher CKD risk compared to the Excellent group (*p* = 0.004). Moreover, a significant interaction was noted between age group and CKD risk within the Poor group (p for interaction = 0.003), and the highest education attainment and CKD risk (p for interaction <0.05).

**Table 4:** Stratified analysis of association between childhood self-reported health status and adulthood CKD risk.

Variables	Fair		Poor	
	OR(95%CI)	*p-*value	OR(95%CI)	*p-*value
Age group				
45–54	1.26(1.00–1.59)	0.050	1.44(0.91–2.28)	0.116
55–64	1.03(0.82–1.29)	0.792	1.74(1.20–2.54)	0.004
65–74	1.08(0.88–1.33)	0.462	0.93(0.63–1.08)	0.697
75+	1.04(0.78–1.39)	0.792	1.01(0.59–1.72)	0.982
*p* for interaction	0.188		0.003	
Sex				
Male	1.08(0.91–1.29)	0.393	1.22(0.89–1.67)	0.220
Female	1.13(0.96–1.33)	0.143	1.27(0.95–1.70)	0.112
*p* for interaction	0.598		0.793	
Region				
Urban	1.11(0.88–1.41)	0.385	1.12(0.68–1.83)	0.667
Rural	1.10(0.96–1.26)	0.026	1.28(1.01–1.62)	0.041
*p* for interaction	0.967		0.618	
The highest education attainment				
Primary and below	1.12(0.97–1.29)	0.121	1.24(0.96–1.60)	0.103
Junior	1.25(0.97–1.62)	0.080	2.04(1.31–3.17)	0.002
Senior and above	0.91(0.66–1.24)	0.548	0.94(0.49–1.81)	0.860
*p* for interaction	<0.001		0.035	
TCM use				
No	1.15(1.01–1.32)	0.039	1.22(0.95–1.58)	0.121
Yes	0.98(0.78–1.22)	0.836	1.28(0.86–0.91)	0.221
*p* for interaction	0.104		0.829	
BMI category				
Underweight	1.45(0.92–2.27)	0.105	1.59(0.78–3.24)	0.200
Normal	1.03(0.87–1.22)	0.719	1.19(0.89–1.60)	0.227
Overweight	1.19(0.97–1.46)	0.090	1.08(0.70–1.67)	0.741
Obesity	1.00(0.72–1.41)	0.986	1.61(0.87–2.99)	0.128
*p* for interaction	0.820		0.731	
Diabetes				
No	1.08(0.94–1.25)	0.268	1.24(0.96–1.60)	0.102
Yes	1.08(0.84–1.38)	0.542	0.86(0.52–1.41)	0.539
*p* for interaction	0.004		0.804	
Hypertension				
No	1.26(1.05–1.50)	0.012	1.34(0.98–1.85)	0.069
Yes	1.00(0.86–1.17)	0.969	1.16(0.87–1.55)	0.300
*p* for interaction	0.566		0.911	

OR, Odds Ratio; CI, Confidence Interval; TCM, traditional Chinese medicine. All analyses adjusted for marital status and annual household income.

## Discussion

The present study utilized data from Wave 3 of the China Health and Retirement Longitudinal Survey to investigate the association between childhood self-reported health status and the risk of chronic kidney disease (CKD) in adulthood. The analysis revealed that the prevalence of CKD among Chinese adults aged 45 years and older is 11.7%. Furthermore, a self-reported poor health status during childhood was found to be significantly associated with an increased risk of CKD in adulthood. These results suggest that poor health during childhood may constitute a significant risk factor for the development of CKD. Indicating that self-reported childhood health could predicts adult CKD incidence independent of traditional risk factors, and simple self-reported childhood health screening could enhance CKD risk stratification in primary care settings. To mitigate the prevalence and associated burden of CKD, it is essential to prioritize the improvement of general health conditions in children.

The prevalence of chronic kidney disease among Chinese adults has been notably high over recent decades. This study found that in 2015, the prevalence of CKD among Chinese adults was 11.7%, which is marginally higher than the figure reported in earlier research (20). Liyanage et al. reported a CKD prevalence of 9.2% (95% CI: 7.4–11.0) based on a population-based study in China (20). Conversely, Zhang et al., employing a multistage, stratified sampling method to obtain a representative sample from 13 provinces, reported a CKD prevalence of 10.8% (95% CI: 10.2–11.3) in 2010 (21). This figure is closely aligned with the current study’s prevalence rate of 11.7%. The observed discrepancy may be attributable to differences in the definitions of CKD used in the studies. Our study defined CKD based on estimated Glomerular Filtration Rate (eGFR) as per the CKD-EPI equation and self-reported physician-diagnosed kidney disease. In contrast, Zhang et al.’s study defined CKD using eGFR and albuminuria.

This study represents the first investigation into the association between self-reported health status during childhood and the risk of chronic kidney disease in adulthood. After controlling for various covariates, it was found that individuals who reported poor health status in childhood had a 1.24-fold increased risk of CKD compared to those who reported excellent childhood health. This indicates that poor childhood health status is associated with a 24% higher risk of developing CKD in adulthood. Although previous research, including numerous original studies, systematic reviews, and meta-analyses, has identified adverse childhood experiences—including health status—as significant risk factors for a range of health outcomes, including 23 different conditions, the specific relationship between adverse childhood experiences and CKD risk has not been thoroughly explored (15, 16, 22–24).

Several mechanisms may elucidate the association between self-reported childhood health status and the risk of chronic kidney disease (CKD) in later life. Firstly, poor health during childhood could result in a reduced number of nephrons, diminished kidney size, and, in some cases, impaired renal function. Evidence from human autopsy and epidemiological studies indicates that children born preterm often exhibit a lower nephron count, reduced kidney size, and, occasionally, compromised renal function (25, 26). Secondly, individuals with poor childhood health status exhibit a higher prevalence of hypertension and diabetes compared to those with better childhood health (13, 14), and both hypertension and diabetes are significant risk factors for CKD (27–30). Thirdly, poor childhood health may be associated with elevated levels of oxidative stress (31), which is another recognized risk factor for CKD (32, 33). Moreover, developmental origins hypothesis indicated that adverse childhood experiences could change the epigenetic characteristics (34), which had been documented could elevate the risk of chronic kidney disease in adulthood (35).

Furthermore, the present study conducted a series of stratification analyses, revealing that the relationship between childhood self-reported health status and adult CKD risk varies across different age groups. Specifically, the association appears to be stronger in the 54–65 age group compared to other subgroups (*P* for interaction = 0.003). This variability may be attributed to the natural history of CKD and survivorship bias. In the 45–54 age group, there may be a higher proportion of preclinical cases (36). Conversely, in the older age group (above 65 years), a greater number of individuals with CKD may have died due to CKD or related diseases and complications (37).

Although the current study utilized National Wave 3 follow-up data from the Chinese Health and Retirement Longitudinal Survey to investigate the association between self-reported childhood health status and the risk of CKD in adulthood, several limitations must be acknowledged. First, the definition of CKD in this study is based on estimated glomerular filtration rate and self-reported diagnoses by physicians. Self-reported disease information may be subject to information bias, including non-differential misclassification bias. Nonetheless, since the CHARLS survey relies on clinical history records for self-reported disease information, this helps mitigate information bias. Second, residual confounding remains a concern in the observed association, as factors such as birth weight and delivery mode were not adjusted for in the logistic regression model. Third, the cross-sectional design of the study imposes limitations on establishing causality between self-reported childhood health status and the risk of CKD in adulthood. Fourth, the assessment of childhood health conditions via a standardized questionnaire, which relied on participants’ recall of past events, may introduce potential recall bias due to the retrospective nature of data. However. It provides cost-effective longitudinal data on early-life health exposures that are often unavailable in medical records, particularly in low- and middle-income country contexts.

## Conclusion

Chronic kidney disease is prevalent among adults in China, and there is a notable association between self-reported childhood health status and the risk of developing chronic kidney disease in adulthood. This relationship appears to be specific to different age groups.

## Data Availability

The raw data supporting the conclusions of this article will be made available by the authors, without undue reservation.
