# Supplementary material for: Association of childhood health with adulthood chronic kidney disease: results from the China Health and Retirement Longitudinal Study
Source: Front Public Health. 2025 Apr 22;13:1538744. doi: 10.3389/fpubh.2025.1538744 (PMC12052567; doi:10.3389/fpubh.2025.1538744)
Supplement: Supplementary file 1 [file Data_Sheet_1.pdf]

Supplemental material

Association of childhood health with adulthood chronic kidney disease: Results from the China Health and Retirement Longitudinal Study

Table S1 the association between childhood self-reported health condition and adulthood chronic kidney diseases in 1:1 matched case-control population

Health condition	Model 1		Model 2		Model 3	
	OR(95%*CI)*	*P*-value	*OR*(95%*CI)*	*P*-value	*OR*(95%*CI)*	*P*-value
**Good**	Ref.		Ref.		Ref.	
**Fair**	1.01(0.86-1.20)	0.860	1.02(0.86-1.21)	0.818	1.03(088-1.25)	0.834
**Poor**	1.76(1.28-2.43)	<0.001	1.71(1.23-2.38)	0.002	1.72(1.24-2.39)	0.003

Abbreviations: *OR*, Odds ratio; *CI*: confidence interval; Ref., Reference.

Model 1 was a crude model; Model 2 was adjusted for age, sex, smoking, alcohol consumption, physical activity, the highest education level, and Chinese traditional medicine; Model 3 was furthermore adjusted for diabetes, hypertension, BMI, marital status and annual household income.

Table S2 Stratified analysis of association between childhood self-reported health status and adulthood CKD risk in 1:1 matched case-control population

	Fair		Poor	
	OR(95%*CI)*	*P*-value	*OR*(95%*CI)*	*P*-value
Age group				
23-49	0.97(0.50-1.86)	0.918	0.62(0.14-2.76)	0.526
50-59	1.06(0.79-1.44)	0.69	1.81(0.98-3.34)	0.059
60-69	1.02(0.78-1.34)	0.89	3.23(1.83-5.68)	<0.001
70-79	1.14(0.78-1.66)	0.503	1.41(0.70-2.81)	0.336
80+	0.61(0.28-1.35)	0.227	0.12(0.01-1.09)	0.060
p for interaction	0.691		0.301	
Sex				
Male	0.93(0.74-1.18)	0.555	1.31(0.81-2.10)	0.269
Female	1.13(0.89-1.44)	0.308	2.45(1.54-3.90)	<0.001
p for interaction	0.254		0.062	
Region				
Urban	0.76(0.54-1.08)	0.122	1.05(0.53-2.09)	0.873
Rural	1.13(0.93-1.36)	0.226	2.13(1.46-3.11)	<0.001
p for interaction	0.052		0.078	
Education level				
Primary and below	1.03(0.84-1.26)	0.781	1.82(1.23-2.71)	0.003
Junior	0.99(0.68-1.44)	0.952	2.42(1.12-5.23)	0.025
Senior and above	1.06(0.66-1.70)	0.801	1.15(0.43-2.92)	0.824
p for interaction	0.022		0.714	
Chinese traditional medicine				
No	1.07(0.88-1.30)	0.508	1.86(1.27-2.71)	0.001
Yes	0.91(0.64-1.29)	0.61	1.64(0.82-3.30)	0.162
p for interaction	0.443		0.762	
BMI category				
Underweight	1.38(0.72-2.68)	0.333	2.08(0.68-6.30)	0.196
Normal	0.90(0.70-1.14)	0.383	1.71(1.08-2.68)	0.021
Overweight	1.08(0.81-1.45)	0.577	1.54(0.82-2.92)	0.18
Obesity	1.18(0.74-1.90)	0.485	2.76(1.01-7.54)	0.047
p for interaction	0.579		0.680	
Diabetes				
No	0.99(0.82-1.21)	0.954	1.72(1.17-2.53)	0.006
Yes	0.98(0.68-1.43)	0.933	1.61(0.76-3.39)	0.213
p for interaction	0.963		0.873	
Hypertension				
No	1.10(0.85-1.42)	0.468	1.81(1.13-2.91)	0.014
Yes	0.97(0.78-1.21)	0.766	1.80(1.14-2.84)	0.012
p for interaction	0.457		0.983	

Abbreviations: OR, Odds Ratio; CI, Confidence Interval; TCM, traditional Chinese

medicine.
